# *In-silico* design of a multi-epitope vaccine candidate against onchocerciasis and related filarial diseases

**DOI:** 10.1038/s41598-019-40833-x

**Published:** 2019-03-13

**Authors:** Robert Adamu Shey, Stephen Mbigha Ghogomu, Kevin Kum Esoh, Neba Derrick Nebangwa, Cabirou Mounchili Shintouo, Nkemngo Francis Nongley, Bertha Fru Asa, Ferdinand Njume Ngale, Luc Vanhamme, Jacob Souopgui

**Affiliations:** 1 Department of Molecular Biology, Institute of Biology and Molecular Medicine, IBMM, Université Libre de Bruxelles, Gosselies, Belgium; 2 Department of Biochemistry, Faculty of Science, Jomo Kenyatta University of Agriculture and Technology, Juja, Kenya; 3 Department of Biochemistry and Molecular Biology, Faculty of Science, University of Buea, Buea, Cameroon; 4 Department of Microbiology and Parasitology, Faculty of Science, University of Buea, Buea, Cameroon; 5 Department of Public Health and Hygiene, Faculty of Health Science, University of Buea, Buea, Cameroon

## Abstract

Onchocerciasis is a parasitic disease with high socio-economic burden particularly in sub-Saharan Africa. The elimination plan for this disease has faced numerous challenges. A multi-epitope prophylactic/therapeutic vaccine targeting the infective L3 and microfilaria stages of the parasite’s life cycle would be invaluable to achieve the current elimination goal. There are several observations that make the possibility of developing a vaccine against this disease likely. For example, despite being exposed to high transmission rates of infection, 1 to 5% of people have no clinical manifestations of the disease and are thus considered as putatively immune individuals. An immuno-informatics approach was applied to design a filarial multi-epitope subunit vaccine peptide consisting of linear B-cell and T-cell epitopes of proteins reported to be potential novel vaccine candidates. Conservation of the selected proteins and predicted epitopes in other parasitic nematode species suggests that the generated chimera could be helpful for cross-protection. The 3D structure was predicted, refined, and validated using bioinformatics tools. Protein-protein docking of the chimeric vaccine peptide with the TLR4 protein predicted efficient binding. Immune simulation predicted significantly high levels of IgG_1_, T-helper, T-cytotoxic cells, INF-γ, and IL-2. Overall, the constructed recombinant putative peptide demonstrated antigenicity superior to current vaccine candidates.

## Introduction

Human onchocerciasis (river blindness), caused by a parasitic nematode *Onchocerca volvulus*, is transmitted by the repeated bites of infected *Simulium* black flies. It remains one of the most debilitating yet neglected tropical diseases (NTDs)^1^. Recent estimates indicate that approximately 15.5 million people worldwide currently live with onchocerciasis, including 12.2 million people with onchocercal skin disease (OSD) and 1.025 million with vision loss^2^. An additional 172 million at-risk people are reported to be in need of preventive chemotherapy^3^. Although *O. volvulus* infection has generally been regarded as a chronic but non-fatal condition, recent analyses have indicated the parasite can result in human death, predominantly in those already blinded by the parasite^4,5^. More than 99% of infected people live in Africa^6^, and the vast majority of those severely affected by OSD and visual loss live in sub-Saharan Africa or Yemen. Some severely affected people also live in Venezuela and Brazil^2^. The public health concern and socio-economic burden of onchocerciasis has led to the creation of various control programmes^7^, and large-scale control efforts have been ongoing for over 40 years^8^. Onchocerciasis control programmes have been successful in the Americas; the number of endemic foci decreased from 13 in six countries to two in two countries during the 4-year period from 2013–2017. The success of onchocerciasis control programmes in Africa however, has been limited to a few isolated communities^9–11^. The lower degree of success of onchocerciasis control programmes in Africa is due to the multiple challenges faced in this part of the world, including risks associated with mass drug administration (MDA) in the relatively large areas of co-endemicity with loiasis^12^, ivermectin (IVM) resistance reported in some endemic foci^13^, and the logistic/financial burden involved in implementing IVM mass drug administration programs^14^. The disease distribution worldwide, as well as the status of preventive chemotherapy in endemic countries was recently published by the World Health Organization (WHO)^15^. In addition, disease modelling studies have suggested that depending on compliance, therapeutic coverage, and levels of parasite transmission, it may not be possible to achieve onchocerciasis elimination even after 50 years of annual IVM treatments^14^. It has therefore been suggested that implementation of MDA programs using IVM alone will not be sufficient to achieve onchocerciasis elimination^16^ especially in Africa where the disease burden is highest^17^. There is therefore a need for new tools to combat this disease^18–20^. The development of a prophylactic/therapeutic vaccine would be ideal to complement present control strategies.

Several observations support the feasibility of a vaccine approach to combat onchocerciasis. Firstly, studies of human populations have provided evidence that naturally acquired immunity against *O. volvulus* infection can occur in humans. As an example, in regions where onchocerciasis is endemic, 1 to 5% of the population who have been exposed to high rates of infection transmission do not show any clinical manifestation of the disease and are thus regarded as putatively immune individuals (PI)^21^. Similar observations have been reported in cattle that are infected by a related parasite species, *Onchocerca ochengi*^22^. Specific antibody responses against the infective L3 larval stage develop over years of exposure in endemic regions^23^; this likely explains the effective anti-L3 immunity acquired with age^24^, consistent with the concept of concomitant immunity^23^. In addition, immunisation of cattle and mice with irradiated L3 larvae protects against *Onchocerca* infection^22,25^. Finally, immuno-epidemiological evidence has led to the conclusion that the concurrent and predominant transmission of *O. ochengi* by *Simulium damnosum* in sub-Saharan Africa could lead to the protection of humans against onchocerciasis caused by *O. volvulus* (zooprophylaxis)^26^. The Onchocerciasis Vaccine for Africa (TOVA) Initiative was launched in 2015 as a response to the London Declaration on Neglected Tropical Diseases^16^. It was of course not the first call for vaccine development initiatives. The selection of vaccine candidates reported in previous studies included either antigens immunogenic in putatively immune individuals or molecules supposedly important for parasite development^2^. The most recent vaccine development initiatives led to the selection of two candidate vaccine antigens, Ov-RAL-2 and Ov-103, for further clinical development^2^. It has however been reported in the majority of cases that single recombinant antigens used for immunoprophylaxis induced insufficient immunity and thus elicited limited protective effects^27,28^. Thus far, vaccine design has mainly targeted the microfilariae (mf) and L3 larval stages of the parasite’s life cycle. The L3 larval stage, together with the moulting larva (mL3) stage, is where *O. volvulus* infection is established. Thus, an antigen promoting anti-L3 larval immunity would block infection^29^ and result in a preventative vaccine. On the other hand, a vaccine targeted at the mf stage, the parasite stage which ensures transmission to the insect vector and which is associated with most of the pathology, would be both therapeutic and help to prevent transmission but would not eliminate the infection^30^. Most previous studies have focused on the use of either irradiated parasites^31–33^ or single recombinant antigens^34,35^; however, the immune responses generated have been inadequate to warrant their use in the development of an effective protective tool^32^. Alternatively, we propose the development of a multi-epitope vaccine candidate which could lead to the generation of a more potent protective immune response^27^. Multi-epitope vaccine candidates have already been designed for several diseases, including leishmaniasis, cholera, dengue^36–38^, and cancers^39,40^, and their efficacies have been further reported in *Helicobacter, Schistosoma* and cancer^27,41–43^.

This work is an attempt to respond to the need for novel tools to help achieve the onchocerciasis elimination goal. According to studies in human and animal models, immunity to *O. volvulus* mf and L3 is associated with B-cell and T-cell responses in addition to antibody-dependent cellular cytotoxicity (ADCC) mechanisms^21,44–46^. IL-5 and IL-4 are deemed necessary for protection against irradiated L3 larva as elimination of either of these cytokines (using either monoclonal antibodies or knockout mice) significantly reduced the protective effects of vaccination against larval *O. volvulus* in mice models^32^. Immunity to microfilariae in a mouse model has however been demonstrated to be independent of IL-4^47^. Furthermore, mf negativity is associated with IL-5 and IL-13 production indicating the role of CD4^+^ cells in resistance to infection^48^. Studies in mice also suggest that infection with microfilariae first stimulates CD8^+^ T-cell reactivity, which has some detrimental effect on the initial establishment of parasites, and subsequently stimulates a CD4^+^ T-cell response that overwhelms and destroys the resident parasite population^45^. TLR4 involvement has also been reported in immune protective responses^31^. The focus of this work was thus the *in-silico* generation of a potential multi-B/T-epitope subunit vaccine candidate for onchocerciasis based on immuno-informatics analyses of six *O. volvulus* antigens selected from previous immunomics studies. Four of these antigens (OVOC10819, OVOC5395, OVOC11598, and OVOC12235) are expressed mainly during the development of the early stages of the infective-stage larvae, L3, in the human host. The other two proteins selected (OVOC8619 and OVOC7083) are expressed predominantly by the microfilariae. These antigens are recognized by sera from the putatively immune individuals who have never developed a patent infection with microfilaridermia^49^.

To address the cross-protection potential of the designed vaccine candidate, the constituent proteins and predicted epitopes were evaluated for amino acid conservation with homologous proteins in other related parasites selected based on their importance in livestock or humans. These include *Onchocerca ochengi* and *Onchocerca flexuosa* responsible for onchocerciasis in livestock leading to huge financial losses^50^, *Loa loa* which is a human filarial nematode that poses a significant challenge to onchocerciasis control programs^51^, and lastly *Brugia malayi* and *Wuchereria bancrofti* which are responsible for lymphatic filariasis, another debilitating filaria disease^52^.

## Results

### Collection of protein sequences and preliminary analyses

The amino acid sequences for six proteins were retrieved from the WormBase database and used to design a potential multi-epitope vaccine against onchocerciasis. Four L3 larval antigens, OVOC10819, OVOC5395, OVOC11598, and OVOC12235, as well as two microfilarial antigens, OVOC8619 and OVOC7083, were selected based on their reactivities in immunomic assays^2,49^. The functional sequences were obtained for OVOC10819, OVOC11598, and OVOC12235 after cleavage of the signal peptide as predicted by SignalP 4.1. No signal peptides were predicted for the other proteins. Localisation analyses predicted these proteins to be localized either in the nucleus (OVOC8619), cell membrane (OVOC10819), extracellular compartment (OVOC12235), or the cytoplasm (OVOC5395, OVOC7083, and OVOC11598). The functional sequences for the proteins were then subjected to linear B-cell and T-cell epitope prediction. The *Mycobacterium tuberculosis* 50S ribosomal protein L7/L12 (RL7_MYCTU), P9WHE3 was retrieved from the UniProt database and used as an adjuvant for the immune interaction based on its ability to act as an agonist for TLR4^37^. Immune adjuvants are a key requirement in vaccine formulation and play a critical role in enhancing the efficacy of vaccines^53^.

### Linear B-cell epitope prediction

Linear B-cell epitopes of varying residue lengths were predicted using different servers, but recurrent epitopes simultaneously predicted with BepiPred 2.0 and one other epitope prediction server (BCpreds, ABCpreds or SVMTrip) were selected for the final vaccine peptide. Some of the predicted linear B-cell epitopes were also predicted to be T-cell epitopes and used in generating the chimera. Overlapping B-cell and T-cell epitopes have the same serial numbers (Table 1B).

**Table 1:** Protein and epitope conservation in related nematode species.

Protein	Percentage identity				
	*O. ochengi*	*O. flexuosa*	*L. loa*	*B. malayi*	*W. bancrofti*
**A**					
OVOC5395	97.7	91.0	74.8	77.8	81.9
OVOC7083	95.7	97.8	Not found	75.0	73.8
OVOC8619	99.4	94.0	85.8	87.1	91.9
OVOC10819	100	85.2	60.5	56.9	58.3
OVOC11598	99.5	67.9	67.9	32.4	Not found
OVOC12235	100.0	27.7	70.7	69.8	69.2
**B**					
**Protein**	**B-cell epitope**	**CTL epitope**		**HTL epitope**	
OVOC5395	1. DRISCADSSESQTKFD (56)	2. TSASTSSFY (100)		1. SESQTKFDFPSFTYI(33)	
		22. STSRVSDRY (55)		2. YPTLNIGNGMEISAK (47)	
	2. SASTSSFYPTLNIGN (73)	21. LSNPYSDKY (33)			
		20. GTNVENKSY (33)			
OVOC7083	3. SDEIKELRQQQLNESKDDYDTLPDVNH (55)	3. QLNESKDDY (66)		4. LLSITKMLSLSVLLL (0)	
		19. SLKKQKMLY (78)			
OVOC8619	5. PAPRAAARGGADRADVSSFGTLAALGSAGAESELG (86)	6. RSNQANNGY (78)		5. VSSFGTLAALGSAGA (73)	
	6. FPEISANMSVMFANSRSNQANNG (52)	6. ISANMSVMF (22)		6. ANMSVMFANSRSNQA (53)	
OVOC10819	7. VAESSKMDHVDVNNVE (38)	18. YSNVIDLRY (55)		8. SESSPFIQQHKTTII (25)	
	9. ICEAETRNNRPAATEV (25)	17. CSDHTQNIL (78)			
	8. GEKNLNLSESSPFIQQ (31)				
OVOC11598	10. SNPMLQASSVEQAPAA (19)			11. RRVRKMVLPPSRGEE (33)	
	11. SRGEEVRKPPSSTDGYESENVESYGQKGV (11)	11. YESENVESY (11)			
OVOC12235	12. LSGYRCTSSIQCQTIIPGSY (55)	16.CTVGYFCEY (100)		12. CTSSIQCQTIIPGSY (47)	
	13. RNDDDISKPKCRNPRA (31)	15. DTPNCVMSY (78)			
	14. SRICPANQIAIGGQ (71)	14. QIAIGGQCY (89)			

(A) Shared identity of the selected proteins in related filarial nematodes. (B) Predicted linear B-cell epitopes and T-cell epitopes selected in order to design the vaccine peptide and their percentage of amino acid identity between the five nematode species is in brackets. The serial numbers assigned to the epitopes indicate the order of positions in the final design of the chimera in Fig. 1.

### Cytotoxic T Lymphocytes (CTL)

A total of 53 CTL (9-mer) ligands were predicted for the six selected proteins using NetCTL 1.2 server set at the default threshold score for epitope identification. From these, 14 epitopes selected either on the basis of their high scores or on their overlap with predicted linear B-cell epitopes were used in generating the chimera (Table 1).

### Helper T Lymphocytes (HTL) epitopes prediction

High-binding MHC-II epitopes for human alleles HLA-DR, HLA-DQ and HLA-DP, predicted with the NetMHCII 2.2 web server based on their IC_50_ scores, were defined as HTL epitopes. A total of eight high-binding HTL epitopes were selected for the final vaccine peptide. Some of the predicted B-cell epitopes overlapped with HTL epitopes (Table 1).

### Protein and epitope conservation in related nematodes

A BLAST search of the selected proteins against the UniProt database revealed a high degree of conservation for all the proteins in related nematodes (ranging from 27.7–100%). Two of the proteins were found to be absent in at most one of the selected nematodes (*Onchocerca ochengi*, *Onchocerca flexuosa, Loa loa, Brugia malayi, Wuchereria bancrofti)*. The greatest percentage conservation was found within the *Onchocerca* species (Table 1A). Multiple sequence alignment of the predicted B-cell and T-cell epitopes also revealed a large degree of conservation across the selected homologous proteins. Epitope percentage identities ranged from 0% to 100%. The epitopes and their percentage identities (in brackets) with homologous proteins in the five other selected nematode species are presented in Table 1B.

### Construction of multi-epitope subunit vaccine

The total number of predicted epitopes used in designing the chimera were 14 linear B-cell epitopes, 14 CTL epitopes, and eight HTL epitopes. Some of the predicted overlapping epitopes were merged to form contiguous sequences (Table 1B). The predicted peptide sequences containing linear B-cell epitopes and T-cell epitopes were fused using GPGPG and AAY linkers. The TLR4 (PDB ID: 4G8A) agonist, 50S ribosomal L7/L12 (Locus RL7_MYCTU) with accession no. P9WHE3, was chosen as an adjuvant and added to the amino terminus of the vaccine peptide using an EAAAK linker in order to potentiate antigen-specific immune responses. In addition, a 6xHis tag was added at the C-terminal to aid in protein purification and identification. The final vaccine peptide generated consisted of 599 amino acid residues derived from 22 merged peptide sequences (Fig. 1).

**Figure 1:**
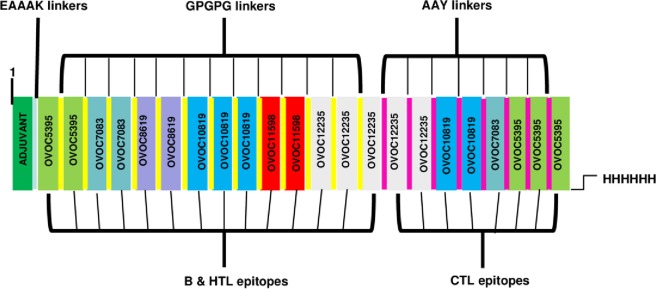
Schematic presentation of the final multi-epitope vaccine peptide. The 599-amino acid long peptide sequence containing an adjuvant (green) at the amino terminal end linked with the multi-epitope sequence through an EAAAK linker (cyan). B epitopes and HTL epitopes are linked using GPGPG linkers (yellow) while the CTL epitopes are linked with the help of AAY linkers (purple). A 6x-His tag is added at the Carboxy terminus for purification and identification purposes.

### IFN-γ inducing epitope prediction

**Figure 2:**
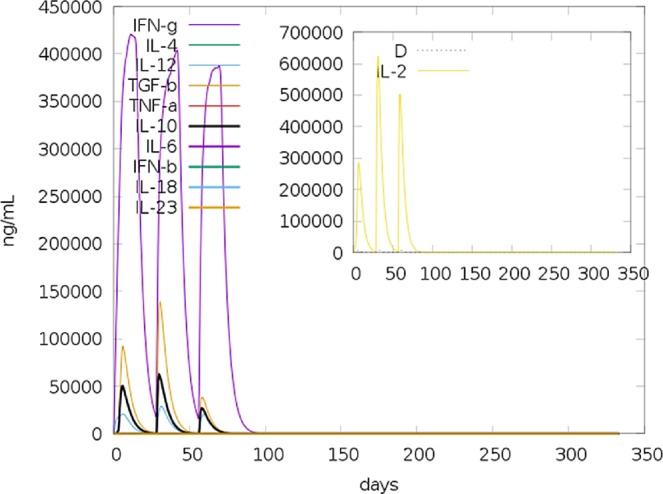
C-ImmSim simulation of the cytokine levels induced by three injections given 4 weeks apart. The main plot shows cytokine levels after the injections. The insert plot shows IL-2 level with the Simpson index, D indicated by the dotted line. D is a measure of diversity. Increase in D over time indicates emergence of different epitope-specific dominant clones of T-cells. The smaller the D value, the lower the diversity.

A total of 127 potential IFN-γ inducing epitopes (15-mer) were predicted for the adjuvant; however, none of these scored above the epitope prediction threshold. On the other hand, a total of 461 potential epitopes (having both negative and positive prediction scores) were predicted for the main vaccine sequence by the IFNepitope server which predicts epitopes using the MERCI software. From the main vaccine sequence a total of 50 IFN-γ inducing epitopes with positive scores were predicted. This prediction was consistent with the simulated level of IFN-γ produced after immunization with the peptide using the C-ImmSim server (http://150.146.2.1/C-IMMSIM/index.php) for immune simulation (Fig. 2).

### Prediction of the antigenicity and allergenicity of the vaccine candidate

The antigenicity of the final sequence (including the adjuvant sequence) was predicted by the VaxiJen 2.0 server to be 0.7428 with a bacteria model and 0.5287 with a parasitic model at a threshold of 0.5 and 0.931486 with ANTIGENpro. The main vaccine sequence (without adjuvant) gave scores of 0.8360 and 0.5920 with bacteria and parasite models respectively at a threshold of 0.5 on VaxiJen 2.0 and 0.937603 on ANTIGENpro. The results indicate that the generated sequences (with and without adjuvant) are both antigenic in nature. The vaccine sequence with and without the adjuvant were both predicted to be non-allergenic on both the AllerTOP v.2 and AllergenFP servers.

### Physiochemical properties and solubility prediction

The molecular weight (MW) of the final protein was predicted to be 62.3 kDa with a theoretical isoelectric point value (pI) of 5.13. The protein is predicted to be slightly acidic in nature based on the pI. The half-life was assessed to be 30 hours in mammalian reticulocytes *in vitro*, and >20 hours in yeast and >10 hours in *E. coli in vivo*. The protein was predicted to be soluble upon expression with a solubility score of 0.755. An instability index (II) of 36.90 was predicted, classifying the protein as stable (II of >40 indicates instability). The estimated aliphatic index was predicted to be 66.91, indicating thermostability^54^. The predicted Grand average of hydropathicity (GRAVY) was −0.407. The negative value indicates the protein is hydrophilic in nature and can interact with water molecules^36^.

### Secondary structure prediction

**Figure 3:**
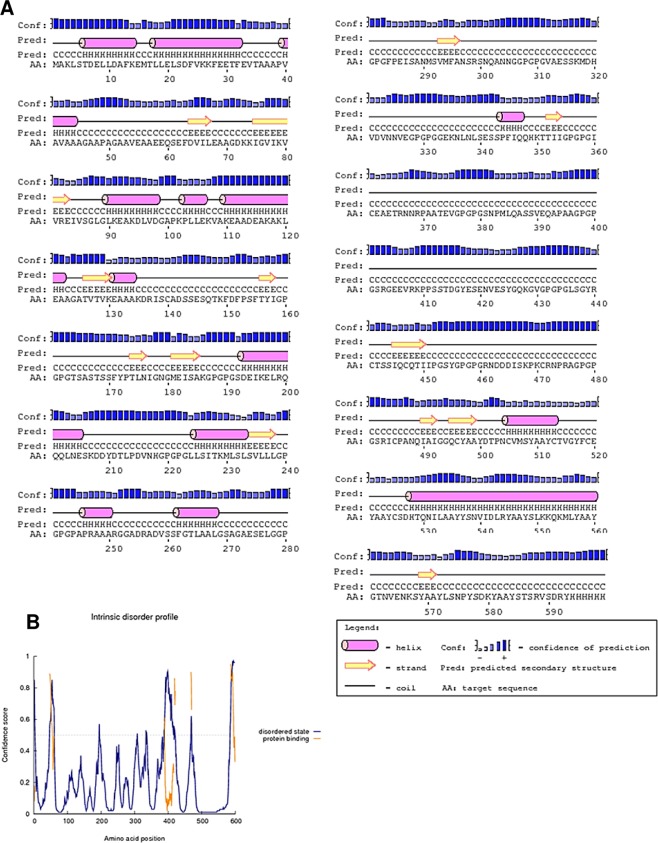
Graphical representation of secondary structure features of the final subunit vaccine sequence. (**A**) The protein is predicted to comprise alpha-helices (22.0%), beta strands (10.0%) and coils (67.0%), and (**B**) 48% of positions are predicted as disordered.

The final chimeric peptide was predicted to contain 22% alpha helix, 10% beta strand, and 67% coil (Fig. 3A). In addition, with regards to solvent accessibility of amino acid residues, 15% were predicted to be exposed, 61% medium exposed, and 22% were predicted to be buried. A total of 292 residues (48%) were predicted to be located in disordered domains by the RaptorX Property server (Fig. 3B). The PSIPRED pictorial prediction of secondary structure and the disorder of the final vaccine sequence is shown below.

### Tertiary structure modelling

**Figure 4:**
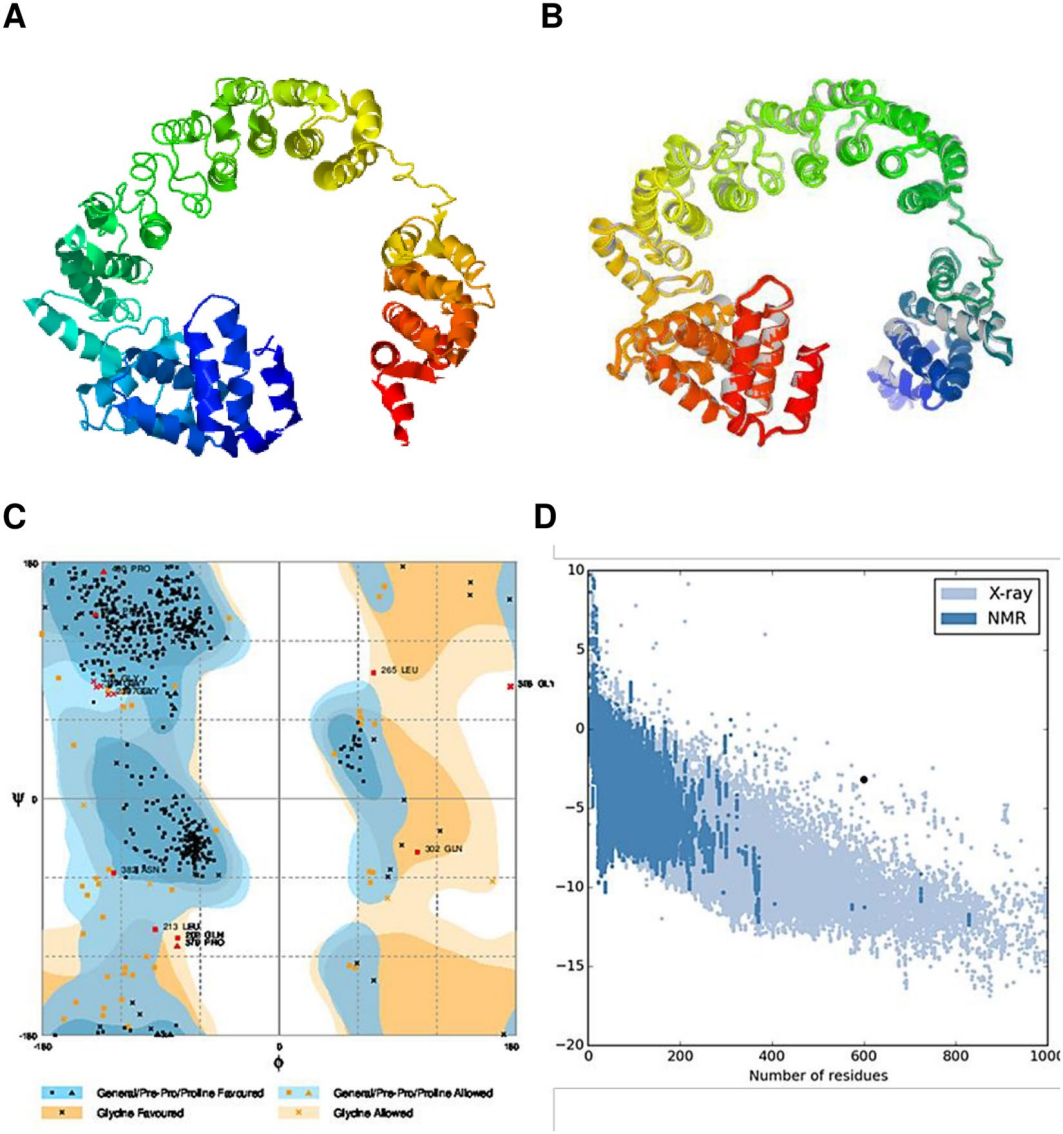
Protein modelling, refinement and validation. (**A**) The final 3D model of the multi-epitope vaccine obtained after homology modelling on I-TASSER. **(B**) Refinement: superimposition of the refined 3D structure (coloured) on the ‘crude model’ (gray) by GalaxyRefine. Validation of the refined model with (**C**) Ramachandran plot analysis showing 94.5%, 4.5% and 1.0% of protein residues in favoured, allowed, and disallowed (outlier) regions respectively and (**D**) ProSA-web, giving a Z-score of −3.45.

The I-TASSER server predicted five tertiary structure models of the designed chimeric protein based on 10 threading templates, of which 1rquA, 5lqwQ, 1dd3A, 2nbiA, and 2ftc were best. All the 10 chosen templates showed good alignment as per their *Z-score* values (ranging from 1.18 to 5.46). The five predicted models had *C-score* values ranging from −3.15 to −0.35. The *C*-score range is typically between −5 and 2, with higher values indicating higher confidence. The model with the highest *C*-score from the homology modelling was selected for further refinement (Fig. 4A). This model had an estimated TM-score of 0.67 ± 0.13 with an estimated RMSD of 8.5 ± 4.5 Å. The TM-score has been proposed as a scale for measuring the structural similarity between two structures^55^. The TM-score was proposed to overcome the problem of RMSD, which is sensitive to local error. A TM-score >0.5 indicates a model of correct topology, and a TM-score <0.17 means random similarity. These cut-off values are independent of protein length.

### Tertiary structure refinement

Refinement of the initial “crude” vaccine model on the ModRefiner server followed by further refinement on the GalaxyRefine server yielded five models. Based on model quality scores for all refined models, model 2 was found to be best based on various parameters including GDT-HA (0.9215), RMSD (0.495), and MolProbity (2.091). The clash score was 14.4, poor rotamers score was 0.2, and Ramachandran plot score was 94.5%. This model was chosen as the final vaccine model for further analysis (Fig. 4B).

### Tertiary structure validation

The Ramachandran plot analysis of the modelled protein revealed that 94.5% of residues in the protein are in favoured regions. This is consistent with the 94.5% score predicted by the GalaxyRefine analysis. Additionally, 4.5% of the residues were predicted to be in allowed regions and only 1.0% in disallowed region (Fig. 4C). The quality and potential errors in the crude 3D model were verified by ProSA-web and ERRAT. The chosen model after refinement had an overall quality factor of 87.9% with ERRAT (not shown) while ProSA-web gave a Z-score of −3.45 (Fig. 4D) for the input vaccine protein model. The protein fell outside the score range commonly found for native proteins of comparable size.

### Conformational B-cell epitopes prediction

A total of 324 residues were predicted to be located in sixteen discontinuous B-cell epitopes with scores ranging from 0.561 to 0.875. The conformation epitopes ranged in size from five to 40 residues.

### Molecular docking of subunit vaccine with immune receptor (TLR4)

**Figure 5:**
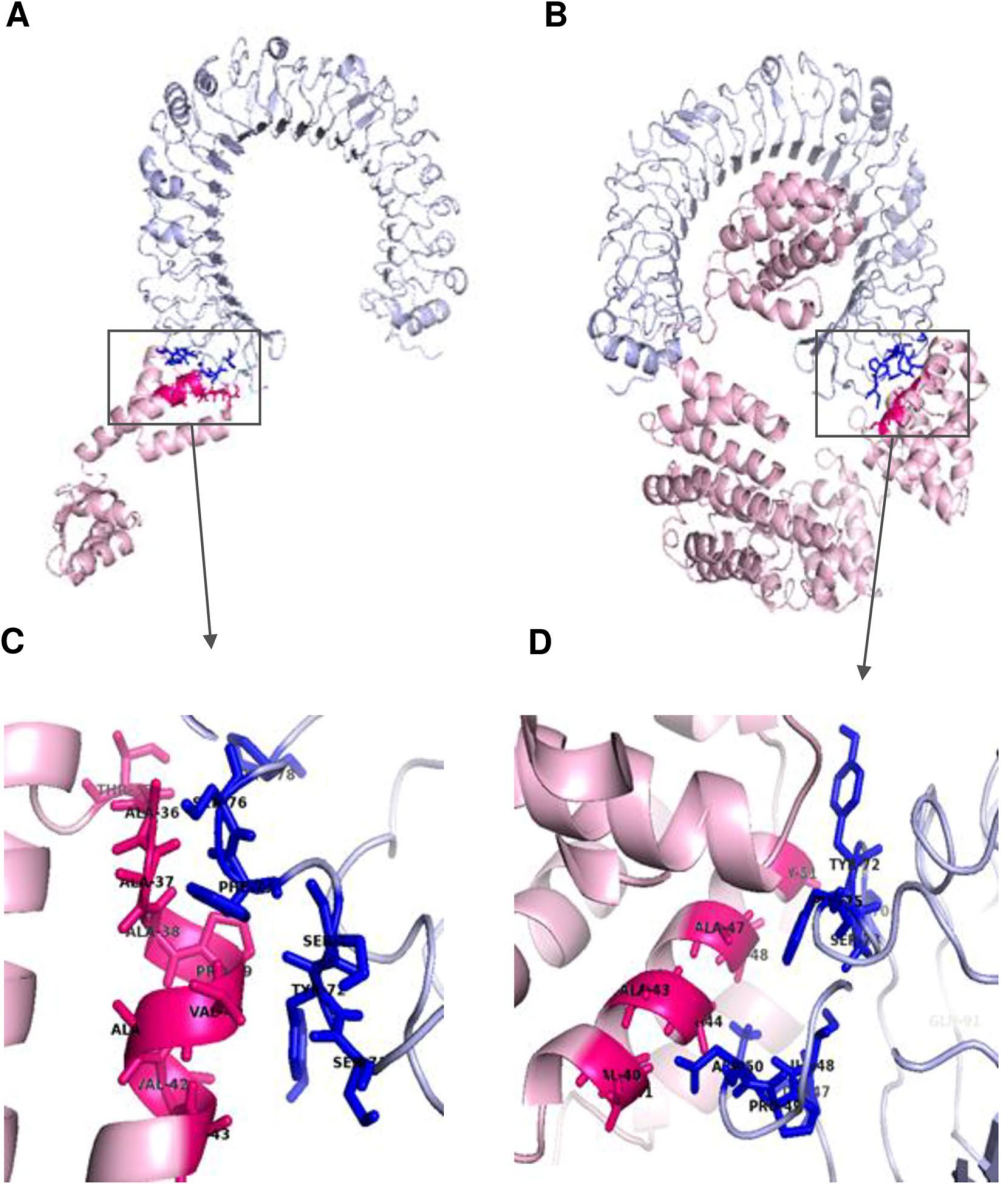
Molecular docking of subunit vaccine with immune receptor (TLR4). (**A**) Docked complexes for adjuvant-TLR4 complex with adjuvant colored light pink, chain B of TLR4 colored light blue and the interface colored hot pink and blue, and (**B**) chimeric protein-TLR4 complex with protein colored light pink, B chain of TLR4 colored light blue and the interface colored in blue and hot pink. (**C**) Interface active residues for adjuvant-TLR4 complex with adjuvant active residues colored hotpink and TLR4 active residues colored in blue, and (**D**) chimeric protein-adjuvant complex with protein active residues colored hotpink and TLR4 active residues colored blue.

The CASTp server was used to determine the protein binding and hydrophobic interaction sites on the protein surface. A binding pocket was identified between residue one and 298 that could act as a potential binding site for TLR4. The molecular surface area of the pocket was 325.1 Å^2^ with a molecular surface volume of 581.3 Å^3^, the mouth molecular area was about 197.8 Å^2^, and the molecular circumference sum was 80 Å. The immune response of TLR-4 against vaccine construct was estimated by analysing the overall conformational stability of vaccine protein-TLR4 docked complex with respect to that of adjuvant-TLR4 docked complex after performing data-driven docking of the complexes. The following active interface amino acid residues: T35, A36, A38, P39, V40, A41, V42, A43, A44, A45, G46, A47, A48, P49 from the adjuvant; T35, A36, A37, A38, V40, A41, A43, A44, A47, A48, G51 from the adjuvant part of the chimeric protein and I48, D50, F54, Y72, S73, F75, S76, S100 from the B chain of TLR4 were predicted and used to ‘drive’ the docking. The top-ranking poses for each docked complex with the lowest total intermolecular energies (chimeric protein-TLR4 complex (−355.25 Kcal/mol), adjuvant-TLR4 complex (−90.48 Kcal/mol)) were selected from HADDOCK clusters with the least average pairwise backbone RMSD (chimeric protein-TLR4 complex (1.1 Å), adjuvant-TLR4 complex (0.8 Å)) at the interface. The relative binding free energies (ΔG) of protein-TLR4 complex (−45.31 Kcal/mol) and adjuvant-TLR4 complex (−17.5 Kcal/mol) suggests that the linking of the chimeric protein to the adjuvant elicits conformational changes that favours stimulation of the TLR4 receptor. Consistently, the number of contacts made at the interface (IC) per property (ICs charged-charged:14, ICs polar-polar: 4, ICs apolar-apolar: 23) for the vaccine protein-TLR4 complex were more than those (ICs charged-charged:3, ICs polar-polar: 1, ICs apolar-apolar: 21) made at the interface of the adjuvant-TLR4 complex. Based on input data used to drive docking, further examination of interface residues common to both complexes were performed as a validation of protocols used for their selection. A41 and A43 of the adjuvant sequences (free adjuvant and adjuvant section of chimeric protein) were involved in interactions at both interfaces, with three distinct residues (D50, Y72 and F75) on the TLR4 B chain. These data not only validate selected docked complexes but may also explain the positive conformational shift on the adjuvant structure (A41 and A43 both interact with D50 of TLR4, in the chimeric protein-TLR4 complex while in the adjuvant-TLR4 complex, A41 and A43 respectively interact with F75 and Y72) resulting from binding of the chimeric protein (Fig. 5).

### Codon optimization of the final vaccine construct

**Figure 6:**
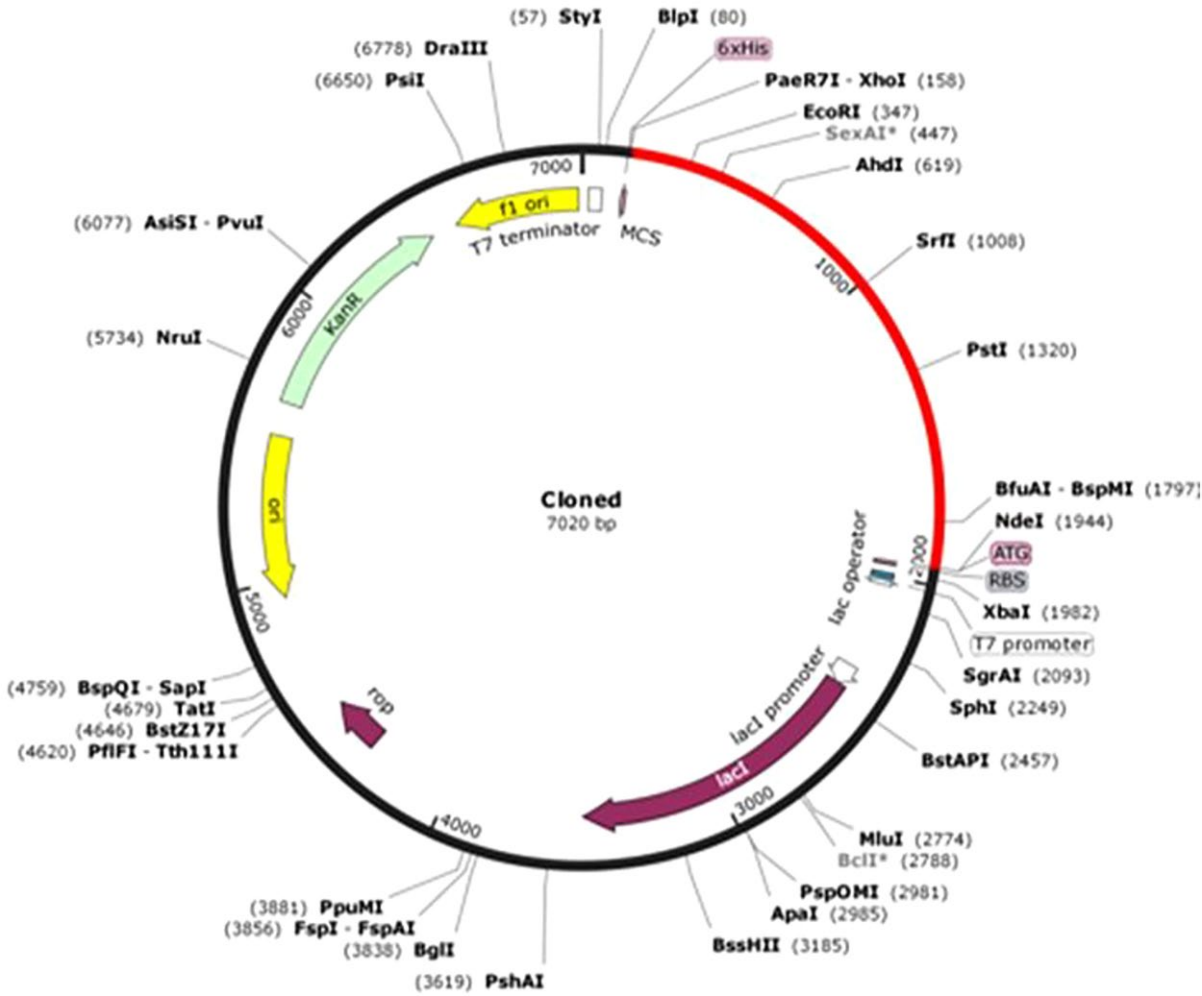
*In silico* restriction cloning of the final vaccine sequence into the pET30a (+) expression vector where the red part represents the gene coding for the vaccine and the black circle represents the vector backbone. The His-tag is located at the Carboxy-terminal end.

In order to optimize codon usage of the vaccine construct in *E. coli* (strain K12) for maximal protein expression, the Java Codon Adaptation Tool (JCat) was used. The length of the optimized codon sequence was 1,797 nucleotides. The Codon Adaptation Index (CAI) of the optimized nucleotide sequence was 0.98, and the average GC content of the adapted sequence was 52.3% showing the possibility of good expression of the vaccine candidate in the *E. coli* host. The optimal percentage range of GC content is between 30% and 70%. Finally, the sequence of the recombinant plasmid was designed by inserting the adapted codon sequences into pET30a (+) vector using SnapGene software (Fig. 6).

### Immune simulation

**Figure 7:**
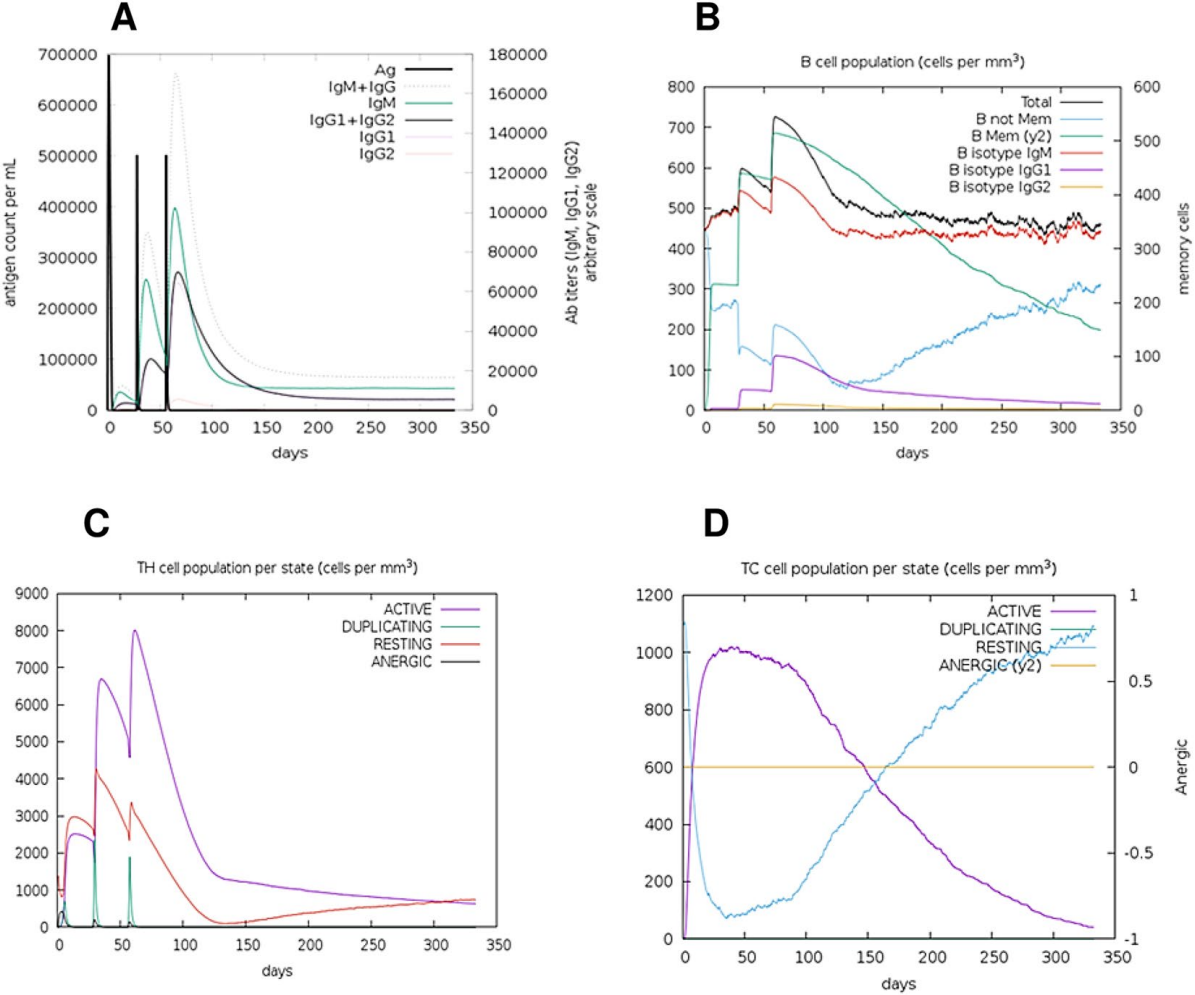
C-ImmSim presentation of an *in silico* immune simulation with the chimeric peptide. (**A**) Immunoglobulin production in response to antigen injections (black vertical lines); specific subclasses are indicated as coloured peaks. (**B**) The evolution of B-cell populations after the three injections. (**C**) The evolution of T-helper, and (**D**) T-cytotoxic cell populations per state after the injections. The resting state represents cells not presented with the antigen while the anergic state represents tolerance of the T-cells to the antigen due to repeated exposures.

C-ImmSim server immune simulation yielded results consistent with actual immune responses as evidenced by a general marked increase in the generation of secondary responses. The primary response was characterized by high levels of IgM. The secondary and tertiary responses saw marked increases in B-cell populations and in levels of IgG_1_ + IgG_2_, IgM, and IgG + IgM antibodies with a corresponding decrease in antigen concentration (Fig. 7A,B). This profile indicates development of immune memory and consequently increased clearance of the antigen upon subsequent exposures (Fig. 7C). A similarly high response was seen in the T_H_ (helper) and T_C_ (cytotoxic) cell populations with corresponding memory development (Fig. 7C,D). Repeated exposure with 12 injections (given four-week apart) triggered increasing levels of IgG_1_ and decreasing levels of IgM while IFN- γ concentration and T_H_ cell population were maintained at high levels throughout the duration of exposure. The chimera without the RL7_MYCTU adjuvant elicited a slightly stronger immune response than the chimera with the adjuvant (data not shown), further supporting the intrinsic antigenicity properties mentioned earlier.

## Discussion

It is estimated that more than two billion people, almost 30% of the world’s population, suffer from at least one NTD, including for example leishmaniasis, lymphatic filariasis, onchocerciasis, schistosomiasis, and soil-transmitted helminthiasis (STHs)^56^. Together, these aforementioned clinical conditions are considered the most common infections in low- and middle-income countries, where they produce a level of global disability and human suffering equivalent to that caused by human immunodeficiency virus/acquired immunodeficiency syndrome (HIV/AIDS) and malaria^57^. Though chemotherapeutic measures do exist for many of these diseases, they are not sustainable countermeasures on their own due to rates of reinfection, risk of drug resistance, and inconsistent maintenance of drug treatment programs. Preventative and/or therapeutic NTD vaccines are needed as long-term solutions^18,58^. Lustigman *et al*. (2017) highlighted the need for a vaccine “now” [2] in order to achieve onchocerciasis elimination^59^. Many vaccines against onchocerciasis, including subunit and whole-organism vaccines, have been tested in animal models^2,28,60–62^; however, none have been translated for use in humans. The focus has recently shifted towards the development of subunit vaccines as they are associated with better safety profiles and are logistically more feasible^63^. Epitope-based vaccines represent a novel approach for generating a specific immune response and avoiding responses against other unfavourable epitopes (like epitopes that may drive immunopathogenic or immune modulating responses) in the complete antigen^64^. Potential advantages of epitope-based vaccines also include increased safety, the opportunity to rationally engineer the epitopes for increased potency and breadth, and the ability to focus immune responses on conserved epitopes^43^.

This work therefore focussed on the *in silico* design and development of a potential multi-epitope vaccine peptide for onchocerciasis using six proteins expressed in one of two parasite stages (the microfilarial and infective larval stages). These stages are preferentially targeted in vaccine development approaches, as any vaccine generated may have both prophylactic and therapeutic potentials. The potential for cross-protection also exists as the selected proteins and predicted epitopes used in generating the chimeric peptide exhibited considerable conservation across the related nematodes analysed. Cross-protection following vaccination with either purified antigens or whole attenuated parasites has been reported in the literature^26,62,65^. The proteins that we selected had exhibited potential to be vaccine candidates in immunomic studies^49^.

It has been reported that immunity to onchocerciasis is dependent on both B- and T-cells^33,45^. The role for antibody-dependent cell-mediated cytotoxicity^46,66,67^ and the TLR4 pathway in immunity to onchocerciasis has also been documented^31^. The potential role of TLR in mediating interactions between helminth parasites and the host immune system has been described^68^. Many filarial parasites (including *O. volvulus*) indeed harbour a *Wolbachia* endosymbiont which can interact with the innate immune system through TLR2 and TLR4^69^. It has been reported that signalling through TLR in filarial nematodes is associated with the major surface protein of *Wolbachia* (wsp)^70^. In the same study, Brattig *et al*. also showed that wsp induced an inflammatory response evidenced by proinflammatory cytokine expression in murine macrophages and DCs stimulated through a TLR2- andTLR4-dependent mechanism. Mice deficient in either of these TLRs failed to elicit the same response. Studies have reported reduced expression of TLR1, 2, 4, and 9 on B-cells, both at the protein and mRNA levels, in subjects infected with lymphatic filariasis compared to uninfected controls. It was thus concluded that down-regulation of TLR1, 2, 4, and 9 appears to be an important mechanism of immune evasion in filarial infections^69^. It has been demonstrated in a murine model of *T. cruzi* infection that TLR4 signalling is required for optimal production of IFN-gamma, TNF-alpha, and nitric oxide (NO) in the spleen of infected animals. TLR4(−/−) mice display higher levels of parasitaemia^71^. We first predicted B-cell and T-cell epitopes from the selected proteins and fused them using appropriate linkers in order to generate a multi-epitope peptide. Vaccine design is improved through the use of specialized spacer sequences^27^. Previously reported AAY and GPGPG linkers^36,37^ were incorporated between the predicted epitopes to produce sequences with minimized junctional immunogenicity, therefore allowing the rational design construction of a potent multi-epitope vaccine^27^. The EAAAK linker, previously reported for bifunctional proteins^72^, was also added between the adjuvant sequence and the fused epitopes in order to reach a high level of expression and improved bioactivity of the fusion protein. Immuno-informatics analyses of the generated vaccine candidate indicated that it contains a large number of high-affinity MHC Class I, MHC Class II, and linear B-cell epitopes. Additionally, interferon-γ (IFN-γ) epitopes were predicted in the final vaccine peptide. It has been reported that IFN-γ secretion may help to control *O. volvulus* infection and results in amicrofilaridermic individuals^73^. The lack of allergenic properties of the designed protein chimera further strengthens its potential as a vaccine candidate. To further assess the potential of the vaccine peptide developed through our work, its antigenicity was compared with that of Ov-RAL-2 and Ov-103. The latter are reported to be lead vaccine candidates for onchocerciasis^74^. The multi-epitope peptide we generated showed higher scores than Ov-RAL-2 and Ov-103 both on Vaxijen v2.0 and ANTIGENpro. Strikingly, the protein chimera we generated gave an antigenic score almost twice that of Ov-103 on ANTIGENpro (0.93 for the chimeric peptide and 0.59 for Ov-103). Vaccines carrying multiple epitopes are often poorly immunogenic and require coupling to adjuvants^27^; however, the designed protein showed similar antigenicity scores with and without the addition of an adjuvant sequence. The absence of the TLR4 agonist sequence even led to slightly higher predicted antigenicity. These results suggest that it may be worthwhile to express the chimeric peptide without the adjuvant used here and assess it in various tests with other adjuvants. Different adjuvants used in immunoprophylactic studies in onchocerciasis have been reported to influence the nature of the elicited immune response^74,75^. Finally, the stability profile of the chimeric peptide was better than Ov-RAL-2 and Ov-103. Ov-RAL-2 was predicted to be unstable.

The molecular weight of our vaccine candidate is 62.3 kDa and it was predicted to be soluble upon expression: this is consistent with its simulated immunogenicity. The solubility of the overexpressed recombinant protein in the *E. coli* host is one of the essential requirements of many biochemical and functional investigations^37^. The theoretical pI is predicted to be 5.13 indicating that the protein is acidic in nature. Also, the predicted instability index indicates that the protein will be stable upon expression, thus further strengthening its potential for use. The aliphatic index showed that the protein contains aliphatic side chains, indicating potential hydrophobicity. All these parameters indicate the recombinant protein is thermally stable and therefore suited for use in endemic areas, most of which are found in sub-Saharan Africa. The knowledge of secondary and tertiary structures of the target protein is essential in vaccine design^27^. Secondary structure analyses indicated that the protein consisted predominantly of coils (67%), with 48% of residues disordered. Natively unfolded protein regions and alpha-helical coiled coils peptides have been reported to be important forms of “structural antigens”. These two structural forms, when tested in synthetic peptides, have the ability to fold into their native structure and thus be recognized by antibodies naturally induced in response to infection^76^. The 3D structure of the vaccine candidate improved markedly after the refinement and showed desirable properties based on Ramachandran plot predictions. The Ramachandran plot shows that most of the residues are found in the favoured and allowed regions (99.0%) with very few residues in the outlier region; this indicates that the quality of the overall model is satisfactory.

Previous studies have reported the involvement of TLR4 in immune protection against *O. volvulus* and other pathogens^31,69,77^. To assess potential immune interaction between TLR4 and the chimeric vaccine peptide, a data-driven protein-protein docking analysis was performed since a TLR4 agonist was used as an adjuvant in the designed chimera. Though, molecular dynamic simulation is more appropriate to predict protein-protein binding efficiency, this option was not chosen because the interface between the TRL4 and the adjuvant was already reported^36^. Our findings support the idea that this interface is not perturbed upon fusion of the adjuvant to our polypeptide. Moreover, the binding energies obtained from the adjuvant alone and the adjuvant-chimera interactions with TLR4 indicated that our designed chimera could indeed have a higher stimulation effect. *In vitro*, purified TLR4 and the recombinant protein could be investigated for such direct interaction. *In vivo*, this chimeric protein is therefore expected to interact with the TLR4 on professional antigen presenting cells, eliciting a potentially protective immune response.

Immune simulation showed results consistent with typical immune responses. Following repeated exposure to the antigen, there was a general increase in the generated immune responses. In onchocerciasis, IgG_1_, IgG_3_ and IgE responses to parasite antigens are implicated in disease protection^24,46,78^. The development of memory B-cells and T- cells was evident, with memory in B-cells lasting several months. Helper T cells were particularly stimulated. Another interesting observation was that levels of IFN-γ and IL-2 rose after the first injection and remained at peak levels following repeated exposures to the antigen. This indicates high levels of T_H_ cells and consequently efficient Ig production, supporting a humoral response. The Simpson index, D for investigation of clonal specificity suggests a possible diverse immune response. This is plausible considering the generated chimeric peptide is composed of several B and T epitopes. It is not yet clear how CD8^+^ T cells may be involved in host protective immunity to *Onchocerca* microfilariae^45^. This may however be a response to stimulation by Th1 and Th2 cells^79^ as cytokines secreted from both cells types (including IL-2 and IL-4) have been reported to be involved in the development of primary and long-lived memory CD8^+^ responses^80,81^. In addition, interferon-γ (IFNγ)-dominant T_H_1-type responses, also reported in putative immune individuals^82,83^, are associated with increased numbers of T_H_1 cells, cytotoxic CD8^+^ T cells, neutrophils, and macrophages^84^.

One of the first steps in validating a candidate vaccine is to screen for immunoreactivity through serological analysis^85^. This requires the expression of the recombinant protein in a suitable host. *Escherichia coli* expression systems are the preferred choice for the production of recombinant proteins^86,87^. In order to achieve high-level expression of our recombinant vaccine protein in *E. coli* (strain K12), codon optimization was carried out. Both the codon adaptability index (0.98) and the GC content (52.3%) were favourable for high-level expression of the protein in bacteria. The next step projected at the moment, is to express this peptide in a bacterial system and perform the various immunological assays needed to validate the results obtained here through immuno-informatics analyses.

## Conclusion

The elimination of onchocerciasis will not be achieved without novel control methods^16^. These involve diagnostic and chemotherapeutic tools as well as a vaccine if possible^2^. In this study, immuno-informatics tools were employed to design a potential vaccine peptide coding for multiple B-cell and T-cell (HTL and CTL) epitopes. Given that the proteins containing these epitopes are expressed in the microfilariae and infective stages of the parasite, the vaccine peptide could potentially provide both prophylactic and therapeutic benefits. This chimeric vaccine peptide could potentially be used as complementary tool to achieve onchocerciasis elimination. As a result of the cross-protection expected from the engineered vaccine candidate, other NTD control programs focusing on related filarial diseases such as lymphatic filariasis and loiasis, as well as control programs for onchocerciasis in livestock, could benefit from this initiative.

## Methodology

### Protein selection for vaccine preparation

The complete amino acid sequences of the six *Onchocerca volvulus* proteins (OVOC10819, OVOC5395, OVOC11598, OVOC12235, OVOC8619, and OVOC7083) were retrieved from WormBase (http://www.wormbase.org/) in the FASTA format. These proteins, which are highly expressed in the microfilariae or L3 larval stages of the parasite, were selected based on previous reports of reactivity with serum from putative immune individuals^2^. The proteins were then subjected to signal peptide analyses using SignalP 4.1 server (http://www.cbs.dtu.dk/services/SignalP/) to discriminate classical secretory and non-secretory proteins. SignalP integrates a prediction of cleavage sites and signal/non-signal peptide prediction based on a combination of several artificial neural networks^88^; it was reported to outperform other predictors in benchmark tests^89^. Localisation predictions were conducted using DeepLoc (http://www.cbs.dtu.dk/services/DeepLoc/), a template-free algorithm that uses deep neural networks to predict protein subcellular localization using only sequence information, achieving good accuracy^90^.

### Linear B-cell epitope prediction

B-cell epitopes are antigenic determinants recognized by the immune system and represent the specific piece of the antigen to which B lymphocytes bind. These play a vital role in vaccine design. Linear B-cell epitopes were predicted predominantly using the BepiPred-2.0 web server (http://www.cbs.dtu.dk/services/BepiPred/). BepiPred-2.0 is based on a random forest algorithm trained on epitopes annotated from antibody-antigen protein structures. This method was superior to other available tools for sequence-based epitope prediction with regards to both epitope data derived from solved 3D structures and a large collection of linear epitopes downloaded from the IEDB database^91^. In addition, the proteins were subjected to linear epitope prediction using three other selected servers. Initially, epitopes for 14-mers were predicted using ABCpred, with a default threshold of 0.51 based on recurrent neural network (http://www.imtech.res.in/raghava/abcpred/). SVMTriP, a Support Vector Machine method to predict linear antigenic epitopes which combines the Tri-peptide similarity and Propensity scores (SVMTriP), was also applied to epitope prediction. Application of SVMTriP to non-redundant linear B-cell epitopes extracted from IEDB achieved a sensitivity of 80.1% and a precision of 55.2% with a five-fold cross-validation. The AUC value for SVMTrip is reported to be 0.702. The combination of similarity and propensity of tri-peptide sub-sequences was reported to improve the prediction performance for linear B-cell epitopes (http://sysbio.unl.edu/SVMTriP/)^92^. Finally, the BCPreds (http://ailab.ist.psu.edu/bcpred/) server, a novel method for predicting linear B-cell epitopes using kernel methods, was applied. The predictive performance of BCPreds (AUC = 0.758) and its output performance is based on SVM as well as the implementation of AAP (Amino acid pair antigenicity; AUC = 0.7)^93^. The use of multiple tools in epitope prediction has been reported to improve the rate of true positives^94^.

### Cytotoxic T lymphocytes (CTL) epitope prediction

CytotoxicT lymphocyte (CTL) epitopes for proteins selected were predicted using the freely-accessible NetCTL 1.2 server (http://www.cbs.dtu.dk/services/NetCTL/). The prediction method integrates prediction of MHC class I binding peptides, proteasomal C-terminal cleavage, and TAP (Transporter Associated with Antigen Processing) transport efficiency. Though the server allows for predictions of CTL epitopes restricted to 12 MHC class I supertypes, only the A1 supertype was used for this work. MHC class I binding and proteasomal cleavage are performed using artificial neural networks. TAP transport efficiency is predicted using a weight matrix^95^. The threshold value for epitope identification was set at 0.75 (default score) for the prediction of CTL epitopes.

### Helper T-cell (HTL) epitope prediction

The NetMHCII 2.2 Server (http://www.cbs.dtu.dk/services/NetMHCII/) was used to predict HTL epitopes of 15-mer length for human alleles. The NetMHCII 2.2 server predicts binding of peptides to HLA-DR, HLA-DQ, and HLA-DP alleles using artificial neuron networks^96^. The prediction of MHC II epitopes was based on receptor affinity, which can be inferred from the IC_50_ values and percentile ranks assigned to each predicted epitope. High-affinity peptides should have IC_50_ values <50 nM. An IC_50_ value <500 nM indicates intermediate affinity, while values <5000 nM indicate low affinity^37^. The percentile rank may therefore be inversely related to the affinity of the epitope and directly related to the IC_50_.

### Protein and epitope conservation in related nematodes

To assess the possibility of cross-protection conferred against other related nematode infections by the chosen proteins and predicted epitopes, a BLAST search was conducted on the UniProt database and the percentage identities for homologous relatives of the selected protein were obtained. The percentage conservation of epitopes was calculated for the different predicted epitopes following multiple sequence alignment of homologous proteins in related nematodes. The nematodes selected for comparison included *Onchocerca ochengi, Onchocerca flexuosa, Loa loa, Brugia malayi*, and *Wuchereria bancrofti*. These were selected based on their importance in humans and livestock.

### Construction of multi-epitope vaccine candidate sequence

Selected high-scoring CTLs, high-affinity HTLs epitopes, as well as B-cell epitopes simultaneously predicted by BepiPred-2.0 and another tool, were used to generate the vaccine candidate sequence. It was noted that some sequences could act as both B-cell and T-cell epitopes. The different epitopes were linked together using AAY and GPGPG linkers. Furthermore, a 50 S ribosomal protein L7/L12 (Locus RL7_MYCTU) was chosen as an adjuvant (Accession no. P9WHE3) to improve the immunogenicity of the vaccine and was added at the N-terminal through an EAAAK linker. The sequence of the adjuvant was retrieved from the UniProt database (http://www.uniprot.org/).

### IFN-γ inducing epitope prediction

Interferon gamma (IFN-γ), a cytokine that plays a critical role in adaptive and innate immune responses, stimulates macrophages and natural killer cells and provides a heightened response to MHC antigens. 15-mer IFN-γ epitopes for the designed peptide were predicted using the IFNepitope server (http://crdd.osdd.net/raghava/ifnepitope/scan.php). IFN- γ epitope predictions were carried out separately for the adjuvant and the main vaccine peptide due to restrictions in the number of residues that can be used for prediction by the server. The server constructs overlapped sequences from which the IFN-γ epitopes are predicted. The prediction was performed by a motif and support vector machine (SVM) hybrid approach. The server is based on a dataset which consists of IFN-γ inducing and non-inducing MHC class-II binder, which can activate T-helper cells^97^.

### Antigenicity and allergenicity prediction of the designed vaccine peptide

ANTIGENpro and VaxiJen v2.0 were used to predict the antigenicity of the protein chimera. ANTIGENpro (http://scratch.proteomics.ics.uci.edu/), which uses protein antigenicity microarray data to predict protein antigenicity, was used to generate an antigenicity index. The accuracy of the server using the combined dataset was estimated to be 76% based on cross-validation experiments^98^. The freely accessible VaxiJen 2.0 server (http://www.ddgpharmfac.net/vaxijen/VaxiJen/VaxiJen.html), which is based on auto- and cross-covariance (ACC) transformation of protein sequences into uniform vectors of principal amino acid properties was used to further assess the antigenicity of the multi-epitope vaccine peptide. The prediction of antigenicity using VaxiJen v2.0 is alignment-free, like ANTIGENpro, and is based on various physiochemical properties of the protein^99^.

AllerTOP v2.0 and AllergenFP were used to predict multi-epitope vaccine allergenicity. AllerTOP v2.0 (http://www.ddg-pharmfac.net/AllerTOP) utilizes amino acid E-descriptors, auto- and cross-covariance transformation, and the k nearest neighbours (kNN) machine learning methods for classification of allergens. The accuracy of this method at 5-fold cross-validation has been reported as 85.3%^100^. AllergenFP (http://ddg-pharmfac.net/AllergenFP/) on the other hand is an alignment-free, descriptor-based fingerprint approach to identify allergens and non-allergens. The approach is based on a four-step algorithm. Initially, the protein sequences are described in terms of their properties, including hydrophobicity, size, relative abundance, and helix and β-strand forming propensities. Subsequently, the generated strings which vary in length are converted into vectors of equal length by auto- and cross-covariance (ACC). The vectors were transformed into binary fingerprints and compared in terms of the Tanimoto coefficient. The approach was applied to known allergens and non-allergens and correctly identified 88% of them with a Matthews correlation coefficient of 0.759^101^.

### Physiochemical properties and solubility prediction

Various physiochemical parameters were assessed using the online web server ProtParam (http://web.expasy.org/protparam/)^102^. These parameters included amino acid composition, theoretical pI, instability index, *in vitro* and *in vivo* half-life, aliphatic index, molecular weight, and grand average of hydropathicity (GRAVY) The solubility of the multi-epitope vaccine peptide was evaluated using the PROSO II server (http://mbiljj45.bio.med.uni-muenchen.de:8888/prosoII/prosoII.seam). PROSO II works based on a classifier exploiting subtle differences between soluble proteins from TargetDB and the PDB and notoriously insoluble proteins from TargetDB. The accuracy was 71% at a default threshold of 0.6. When evaluated by 10-fold cross-validation it achieved 71% accuracy (area under ROC curve = 0.785)^103^.

### Secondary structure prediction

PSIPRED and RaptorX Property were used for secondary structure predictions. PSIPRED, a web-based freely-accessible online server (http://bioinf.cs.ucl.ac.uk/psipred/), was used to predict the protein secondary structure for the input of primary amino acid sequences in a precise manner. Position-specific iterated BLAST (Psi-Blast) was performed to identify and select sequences showing significant homology to the vaccine protein. PSIPRED 3.2 accomplished a normal Q3 score of 81.6% when an exceptionally stringent cross-approval strategy was utilized to assess its performance. The selected sequences were utilized to construct a position-specific scoring matrix^37^. The RaptorX Property web server (http://raptorx.uchicago.edu/StructurePropertyPred/predict/), which is a template-free approach, was further employed to predict the secondary structure properties of the protein chimera. The server uses an emerging machine learning model called DeepCNF (Deep Convolutional Neural Fields) to simultaneously predict secondary structure (SS), solvent accessibility (ACC), and disorder regions (DISO)^104^. This server achieved ~84% Q3 accuracy for 3-state SS, ~72% Q8 accuracy for 8-state SS, ~66% Q3 accuracy for 3-state solvent accessibility, and ~0.89 Area Under the ROC Curve (AUC) for disorder prediction^105^.

### Tertiary structure prediction

Homology modelling of the final multi-epitope vaccine peptide was carried out using the I-TASSER server (https://zhanglab.ccmb.med.umich.edu/I-TASSER/). The I-TASSER (Iterative Threading ASSEmbly Refinement) server is an integrated platform for automated protein structure and function prediction based on the sequence-to-structure-to-function paradigm. Starting from an amino acid sequence, I-TASSER first generates three-dimensional (3D) atomic models from multiple threading alignments and iterative structural assembly simulations^106^. I-TASSER has been ranked the best server for protein structure prediction in the last five community-wide CASP experiments^107^.

### Refinement of the tertiary structure

The 3D model obtained for the multi-epitope vaccine peptide was refined in a two-step process, initially using ModRefiner (https://zhanglab.ccmb.med.umich.edu/ModRefiner/) and then the GalaxyRefine server (http://galaxy.seoklab.org/cgi-bin/submit.cgi?type=REFINE). The construction and refinement of protein structures from Cα traces is carried out by the ModRefiner server based on a two-step, atomic-level energy minimization. This results in improvements in both global and local structures, with more accurate side chain positions, better hydrogen-bonding networks, and fewer atomic overlaps^108^. The GalaxyRefine server on the other hand is based on a refinement method that has been successfully tested in community-wide CASP10 experiments. This method initially rebuilds side chains and performs side chain repacking and subsequently uses molecular dynamics simulation to achieve overall structure relaxation. The GalaxyRefine server method performed the best in improving local structure quality according to the CASP10 assessment. This method, when used to refine the models generated by state-of-the-art protein structure prediction servers can improve the quality of both global and local structures^109^.

### Tertiary structure validation

Model validation is a critical step in the model building process as it detects potential errors in predicted 3D models^37^. ProSA-web (https://prosa.services.came.sbg.ac.at/prosa.php) was used initially to achieve this. ProSA-web provides an easy-to-use interface to the program ProSA and is commonly used in protein tertiary structure validation. ProSA calculates an overall quality score for a specific input structure, and this is displayed in the context of all known protein structures. Also, any problematic parts of a structure are shown and highlighted in a 3D molecule viewer. If the calculated score falls outside the range characteristic of native proteins the structure likely contains errors^110^. The ERRAT server (http://services.mbi.ucla.edu/ERRAT/) was also used to analyse non-bonded atom-atom interactions compared to reliable high-resolution crystallography structures. A Ramachandran plot was obtained through the RAMPAGE server (http://mordred.bioc.cam.ac.uk/~rapper/rampage.php). The Ramachandran plot is a way to visualize energetically allowed and disallowed dihedral angles psi (ψ) and phi (φ) of an amino acid and is calculated based on van der Waal radius of the side chain. The results from RAMPAGE include the percentage of residues in allowed and disallowed regions which define the quality of the modelled structure. The server uses the PROCHECK principle to validate a protein structure by using a Ramachandran plot and separates plots for Glycine and Proline residues^111^.

### Discontinuous B-cell epitope prediction

It has been estimated that >90% of B-cell epitopes are discontinuous, i.e. they consist of segments distantly separated in the pathogen protein sequence that are brought into proximity by the folding of the protein^112,113^. ElliPro (http://tools.iedb.org/ellipro/) was used to predict discontinuous (conformational) B-cell epitopes for the validated 3D structure. ElliPro implements three algorithms to approximate the protein shape as an ellipsoid, calculate the residue protrusion index (PI), and cluster neighbouring residues based on their PI values. ElliPro provides a score for each output epitope described as a PI value averaged over each epitope residue. An ellipsoid with a PI value of 0.9 consists of 90% included protein residues while the remaining 10% of residues lie outside of ellipsoids. For each epitope residue, the PI value is calculated based on the centre of mass of residue residing outside the largest possible ellipsoid. In comparison with other structure-based methods used for epitope prediction, ElliPro performed the best and gave an AUC value of 0.732 considering the most significant prediction for each protein^114^.

### Molecular docking of designed chimeric protein with TLR4

The generation of an appropriate immune response is dependent on the interaction between an antigenic molecule and a specific immune receptor. The binding pockets or cavities in the TLR-4 receptor was predicted by using the CASTp server (http://sts.bioe.uic.edu/castp/). In CASTp, voids are defined as buried, unfilled empty spaces inside proteins following removal of all heteroatoms inaccessible to water molecules (modelled as a spherical probe of 1.4 Å) from outside. Pockets on the other hand are defined as concave caverns with constrictions at the opening on the surface regions of proteins. CASTp is excellent at providing identification and measurements of surface accessible binding pockets along with the information of inner inaccessible cavities for protein molecules^115^. Molecular docking of the multi-epitope vaccine peptide with the TLR4 (PDB ID: 4G8A) receptor was performed using the HADDOCK 2.2 web server in order to evaluate the interaction between ligand and receptor and consequently the development of an immune response. Protective immunity against the larval stages of *Onchocerca volvulus* is reported to be dependent on TLR4^31^. Consistently, mice with a natural mutation of TLR4 (C3H/HeJ) show a higher degree of susceptibility to *Litomodoides sigmodontis* infection^77^. TLR4 has also been reported to play a major role in ocular pathogenesis^116^.

In order to predict such interaction, data-driven docking of designed chimeric protein-TLR4 and adjuvant-TLR4 complexes were performed. The adjuvant which is a 50S ribosomal protein with the accession number P9WHE3 has the ability to stimulate TLR4, thus serving as a TLR4 agonist^37^. While information-driven docking can predict interacting subunits and the overall shape of complexes, knowledge about which specific residues involved in the interaction is needed to drive the docking calculations. In this regard, CPORT (https://milou.science.uu.nl/services/CPORT/)^117^ was used to predict active interface residues of chimeric protein (including adjuvant), adjuvant and TLR4. The HADDOCK webserver (version2.2) (http://haddock.science.uu.nl/services/HADDOCK2.2) was then employed to perform docking simulations of the chimeric protein-TLR4 and adjuvant-TLR4 complexes due to the server’s ability to incorporate experimental or bioinformatics data to ‘drive’ the docking and to perform short MD simulations of docked complexes^118,119^. HADDOCK (High Ambiguity Driven DOCKing) consists of a collection of python scripts that makes use of Crystallography and NMR Systems for structure calculation. After proper orientation and docking calculations of the complexes are performed, HADDOCK passes resulting structures into, and invokes a final MD simulation stage consisting of a gentle refinement in an 8 Å shell of TIP3P water molecules. The MD steps (5000) are performed at 300 K with position restraints only on noninterface heavy atoms and a final cooling stage of 1000 MD steps run at 300, 200, and 100 K^120^. Inter- and intramolecular energies are evaluated using full electrostatic and van der Waals energy terms with an 8.5 Å distance cut-off using OPLS nonbonded parameters and the final structures are clustered using the pairwise backbone RMSD at the interface. Lastly, the binding affinities of the top ranking docking pose of chimeric protein-TLR4 complex and adjuvant-TLR4 complex were respectively, predicted using the PRODIGY (PROtein binDIng enerGY prediction) webserver (https://nestor.science.uu.nl/prodigy/)^121^.

### *In silico* cloning optimization of designed vaccine candidate

Reverse translation and codon optimization were performed using Java Codon Adaptation Tool (JCat) server (http://www.prodoric.de/JCat) in order to express the multi-epitope vaccine construct in a selected expression vector^122^. Codon optimization was performed in order to express the final vaccine construct in the *E. coli* (strain K12) host, as codon usage of *E. coli* differs from that of the native host *O. volvulus*, where the sequence of the final vaccine construct is derived. Three additional options were selected to avoid the rho-independent transcription termination, prokaryote ribosome binding site, and restriction enzymes cleavage sites. The JCat output includes the codon adaptation index (CAI) and percentage GC content, which can be used to assess protein expression levels. CAI provides information on codon usage biases; the ideal CAI score is 1.0 but >0.8 is considered a good score^123^. The GC content of a sequence should range between 30–70%. GC content values outside this range suggest unfavourable effects on translational and transcriptional efficiencies^36^. To clone the optimized gene sequence of the final vaccine construct in *E. coli* pET-30a (+) vector, *Nde* I and *Xho* I restriction sites were introduced to the N and C-terminals of the sequence, respectively. Finally, the optimized sequence (with restriction sites) was inserted into the pET-30a (+) vector using SnapGene tool to ensure vaccine expression.

### Immune Simulation

To further characterize the immunogenicity and immune response profile of the chimeric peptide, *in silico* immune simulations were conducted using the C-ImmSim server (http://150.146.2.1/C-IMMSIM/index.php). C-ImmSim is an agent-based model that uses a position-specific scoring matrix (PSSM) for immune epitope prediction and machine learning techniques for prediction of immune interactions. It “simultaneously simulates three compartments that represent three separate anatomical regions found in mammals: (i) the bone marrow, where hematopoietic stem cells are simulated and produce new lymphoid and myeloid cells; (ii) the thymus, where naive T cells are selected to avoid auto immunity; and (iii) a tertiary lymphatic organ, such as a lymph node”^124^. In accordance with the TOVA approach for the target product profile of a prophylactic onchocerciasis vaccine, three injections were given at intervals of four weeks^125^. All simulation parameters were set at default with time steps set at 1, 84, and 168 (each time step is 8 hours and time step 1 is injection at time = 0). Therefore, three injections were given four weeks apart. Furthermore, 12 injections of the designed peptide were given four weeks apart to simulate repeated exposure to the antigen seen in a typical endemic area so as to probe for clonal selection. The Simpson index, D (a measure of diversity) was interpreted from the plot.

## Data Availability

All data generated or analysed during the study are included in the submitted manuscript. The sequences of the protein analysed can be retrieved from WormBase (wormbase.org) and UniProt database (uniport.org) using their accession numbers.

## References

[1] Katabarwa M (2014). Transmission of Onchocerca volvulus by Simulium neavei in Mount Elgon Focus of Eastern Uganda Has Been Interrupted. The American Journal of Tropical Medicine and Hygiene.

[2] Lustigman, S. *et al*. Onchocerca volvulus: The Road from Basic Biology to a Vaccine. *Trends Parasitol* (2017). 10.1016/j.pt.2017.08.011 PMC5748257 28958602

[3] Vlaminck J, Fischer PU, Weil GJ (2015). Diagnostic Tools for Onchocerciasis Elimination Programs. Trends Parasitol.

[4] Walker, M. *et al*. Density-dependent mortality of the human host in onchocerciasis: relationships between microfilarial load and excess mortality. *PLoS Negl Trop Dis* **6** (2012). 10.1371/journal.pntd.0001578 PMC3313942 22479660

[5] Pion SD, Kamgno J, Demanga N, Boussinesq M (2002). Excess mortality associated with blindness in the onchocerciasis focus of the Mbam Valley, Cameroon. Ann Trop Med Parasitol.

[6] Colebunders R (2018). From river blindness control to elimination: bridge over troubled water. Infect Dis Poverty.

[7] Kale OO (1998). Onchocerciasis: the burden of disease. Ann Trop Med Parasitol.

[8] Boatin, B. The Onchocerciasis Control Programme in West Africa (OCP). *Ann Trop Med Parasitol* **102** (2008). 10.1179/136485908X337427 18718148

[9] Diawara, L. *et al*. Feasibility of onchocerciasis elimination with ivermectin treatment in endemic foci in Africa: first evidence from studies in Mali and Senegal. *PLoS Negl Trop Dis* **3** (2009). 10.1371/journal.pntd.0000497 PMC2710500 19621091

[10] Traore, M. O. *et al*. Proof-of-principle of onchocerciasis elimination with ivermectin treatment in endemic foci in Africa: final results of a study in Mali and Senegal. *PLoS Negl Trop Dis* **6** (2012). 10.1371/journal.pntd.0001825 PMC3441490 23029586

[11] Kamga GR (2017). Important progress towards elimination of onchocerciasis in the West Region of Cameroon. Parasit Vectors.

[12] Twum-Danso, N. A. Loa loa encephalopathy temporally related to ivermectin administration reported from onchocerciasis mass treatment programs from 1989 to 2001: implications for the future. *Filaria J* **2** (2003). 10.1186/1475-2883-2-S1-S7 PMC2147656 14975064

[13] Lustigman S, McCarter JP (2007). Ivermectin Resistance in Onchocerca volvulus: Toward a Genetic Basis. PLoS Neglected Tropical Diseases.

[14] Turner, H. C. *et al*. Reaching the london declaration on neglected tropical diseases goals for onchocerciasis: an economic evaluation of increasing the frequency of ivermectin treatment in Africa. *Clin Infect Dis* **59** (2014). 10.1093/cid/ciu467 PMC4166981 24944228

[15] World Health Organisation, W. Onchocerciasis (2018).

[16] Hotez PJ (2015). The Onchocerciasis Vaccine for Africa–TOVA–Initiative. PLoS Negl Trop Dis.

[17] Mackenzie CD, Homeida MM, Hopkins AD, Lawrence JC (2012). Elimination of onchocerciasis from Africa: possible?. Trends Parasitol.

[18] Hotez PJ (2016). Eliminating the Neglected Tropical Diseases: Translational Science and New Technologies. PLoS Negl Trop Dis.

[19] Rebollo MP (2018). Onchocerciasis: shifting the target from control to elimination requires a new first-step—elimination mapping. International Health.

[20] Kim YE (2015). Control, elimination, and eradication of river blindness: scenarios, timelines, and ivermectin treatment needs in Africa. PLoS Negl Trop Dis.

[21] Turaga PS (2000). Immunity to onchocerciasis: cells from putatively immune individuals produce enhanced levels of interleukin-5, gamma interferon, and granulocyte-macrophage colony-stimulating factor in response to Onchocerca volvulus larval and male worm antigens. Infect Immun.

[22] Tchakoute VL (2006). In a bovine model of onchocerciasis, protective immunity exists naturally, is absent in drug-cured hosts, and is induced by vaccination. Proc Natl Acad Sci USA.

[23] MacDonald AJ (2002). Differential Cytokine and Antibody Responses to Adult and Larval Stages of Onchocerca volvulus Consistent with the Development of Concomitant Immunity. Infection and Immunity.

[24] Cho-Ngwa F, Liu J, Lustigman S (2010). The Onchocerca volvulus cysteine proteinase inhibitor, Ov-CPI-2, is a target of protective antibody response that increases with age. PLoS Negl Trop Dis.

[25] Yutanawiboonchai W, Brigandi RA, Rotman HL, Abraham D (1996). Structural and molecular specificity of antibody responses in mice immune to third stage larvae of Onchocerca volvulus. Parasite Immunol.

[26] Achukwi MD, Harnett W, Enyong P, Renz A (2007). Successful vaccination against Onchocerca ochengi infestation in cattle using live Onchocerca volvulus infective larvae. Parasite Immunol.

[27] Meza B, Ascencio F, Sierra-Beltrán AP, Torres J, Angulo C (2017). A novel design of a multi-antigenic, multistage and multi-epitope vaccine against Helicobacter pylori: An in silico approach. Infection, Genetics and Evolution.

[28] Hess JA (2014). Vaccines to combat river blindness: expression, selection and formulation of vaccines against infection with Onchocerca volvulus in a mouse model. Int J Parasitol.

[29] Abraham D, Lucius R, Trees AJ (2002). Immunity to Onchocerca spp. in animal hosts. Trends in Parasitology.

[30] Cook JA, Steel C, Ottesen EA (2001). Towards a vaccine for onchocerciasis. Trends in Parasitology.

[31] Kerepesi LA, Leon O, Lustigman S, Abraham D (2005). Protective immunity to the larval stages of onchocerca volvulus is dependent on Toll-like receptor 4. Infect Immun.

[32] Lange AM, Yutanawiboonchai W, Scott P, Abraham D (1994). IL-4- and IL-5-dependent protective immunity to Onchocerca volvulus infective larvae in BALB/cBYJ mice. J Immunol.

[33] Folkard SG, Taylor MJ, Butcher GA, Bianco AE (1997). Protective responses against skin-dwelling microfilariae of Onchocerca lienalis in severe combined immunodeficient mice. Infection and Immunity.

[34] Taylor MJ, Jenkins RE, Bianco AE (1996). Protective immunity induced by vaccination with Onchocerca volvulus tropomyosin in rodents. Parasite Immunol.

[35] Taylor MJ, Abdel-Wahab N, Wu Y, Jenkins RE, Bianco AE (1995). Onchocerca volvulus larval antigen, OvB20, induces partial protection in a rodent model of onchocerciasis. Infect Immun.

[36] Ali M (2017). Exploring dengue genome to construct a multi-epitope based subunit vaccine by utilizing immunoinformatics approach to battle against dengue infection. Sci Rep.

[37] Khatoon N, Pandey RK, Prajapati VK (2017). Exploring Leishmania secretory proteins to design B and T cell multi-epitope subunit vaccine using immunoinformatics approach. Sci Rep.

[38] Nezafat N (2016). Designing an efficient multi-epitope peptide vaccine against Vibrio cholerae via combined immunoinformatics and protein interaction based approaches. Comput Biol Chem.

[39] Kawashima I (1998). The Multi-epitope Approach for Immunotherapy for Cancer: Identification of Several CTL Epitopes from Various Tumor-Associated Antigens Expressed on Solid Epithelial Tumors. Human Immunology.

[40] Zhang L (2018). Multi-epitope vaccines: a promising strategy against tumors and viral infections. Cell Mol Immunol.

[41] Nezafat N (2015). Production of a novel multi-epitope peptide vaccine for cancer immunotherapy in TC-1 tumor-bearing mice. Biologicals.

[42] Yang W, Jackson DC, Zeng Q, McManus DP (2000). Multi-epitope schistosome vaccine candidates tested for protective immunogenicity in mice. Vaccine.

[43] Zhou W-Y (2009). Therapeutic efficacy of a multi-epitope vaccine against Helicobacter pylori infection in BALB/c mice model. Vaccine.

[44] Lustigman S, MacDonald AJ, Abraham D (2003). CD4+-dependent immunity to Onchocerca volvulus third-stage larvae in humans and the mouse vaccination model: common ground and distinctions. International Journal for Parasitology.

[45] Folkard SG, Bianco AE (1995). Roles for both CD+ and CD8+ T cells in protective immunity against Onchocerca lienalis microfilariae in the mouse. Parasite Immunology.

[46] Abraham D (2004). Immunoglobulin E and Eosinophil-Dependent Protective Immunity to Larval Onchocerca volvulus in Mice Immunized with Irradiated Larvae. Infection and Immunity.

[47] Johnson EH (1998). Immune responses to third stage larvae of Onchocerca volvulus in interferon-gamma and interleukin-4 knockout mice. Parasite Immunol.

[48] Brattig NW (2002). Onchocerca volvulus-Exposed Persons Fail to Produce Interferon-γ in Response to O. volvulus Antigen but Mount Proliferative Responses with Interleukin-5 and IL-13 Production that Decrease with Increasing Microfilarial Density. The Journal of Infectious Diseases.

[49] Bennuru, S. *et al*. Stage-Specific Transcriptome and Proteome Analyses of the Filarial Parasite Onchocerca volvulus and Its Wolbachia Endosymbiont. *MBio* **7** (2016). 10.1128/mBio.02028-16 PMC5137501 27881553

[50] Solismaa M (2008). Filarioid nematodes in cattle, sheep and horses in Finland. Acta Veterinaria Scandinavica.

[51] Kelly-Hope L (2017). Loa loa vectors Chrysops spp.: perspectives on research, distribution, bionomics, and implications for elimination of lymphatic filariasis and onchocerciasis. Parasites & Vectors.

[52] Chakraborty S, Gurusamy M, Zawieja DC, Muthuchamy M (2013). Lymphatic filariasis: Perspectives on lymphatic remodeling and contractile dysfunction in filarial disease pathogenesis. Microcirculation (New York, N.Y.: 1994).

[53] Marciani DJ (2003). Vaccine adjuvants: role and mechanisms of action in vaccine immunogenicity. Drug Discov Today.

[54] Ikai A (1980). Thermostability and aliphatic index of globular proteins. J Biochem.

[55] Zhang Y, Skolnick J (2004). Scoring function for automated assessment of protein structure template quality. Proteins.

[56] Terry FE (2015). Time for T? Immunoinformatics addresses vaccine design for neglected tropical and emerging infectious diseases. Expert Review of Vaccines.

[57] Bethony JM (2011). Vaccines to combat the neglected tropical diseases. Immunol Rev.

[58] Beaumier CM, Gillespie PM, Hotez PJ, Bottazzi ME (2013). New vaccines for neglected parasitic diseases and dengue. Transl Res.

[59] Expanded Special Project for the Elimination of Neglected Tropical Diseases, E. About the NTD Portal. Vol. 2018 (2017).

[60] Babayan SA, Allen JE, Taylor DW (2012). Future prospects and challenges of vaccines against filariasis. Parasite Immunol.

[61] Makepeace BL (2009). Immunisation with a multivalent, subunit vaccine reduces patent infection in a natural bovine model of onchocerciasis during intense field exposure. PLoS Negl Trop Dis.

[62] Townson S, Nelson GS, Bianco AE (1985). Immunity to Onchocerca lienalis microfilariae in mice. II. Effects of sensitization with a range of heterologous species. J Helminthol.

[63] Vartak, A. & Sucheck, S. J. Recent Advances in Subunit Vaccine Carriers. *Vaccines (Basel)* **4** (2016). 10.3390/vaccines4020012 PMC4931629 27104575

[64] Moise L (2015). iVAX: An integrated toolkit for the selection and optimization of antigens and the design of epitope-driven vaccines. Hum Vaccin Immunother.

[65] Griffiths G, Pritchard DI (1994). Vaccination against gastrointestinal nematodes of sheep using purified secretory acetylcholinesterase from Trichostrongylus colubriformis–an initial pilot study. Parasite Immunol.

[66] Johnson EH, Lustigman S, Brotman B, Browne J, Prince AM (1991). Onchocerca volvulus: *in vitro* killing of microfilaria by neutrophils and eosinophils from experimentally infected chimpanzees. Trop Med Parasitol.

[67] Titanji VP, Nde PN, Mbacham WF (1992). Cell-mediated and monoclonal antibody-dependent killing of Onchocerca volvulus microfilariae. Scand J Immunol Suppl.

[68] Rajasekaran S, Anuradha R, Bethunaickan R (2017). TLR Specific Immune Responses against Helminth Infections. Journal of Parasitology Research.

[69] Babu S, Blauvelt CP, Kumaraswami V, Nutman TB (2005). Diminished expression and function of TLR in lymphatic filariasis: a novel mechanism of immune dysregulation. J Immunol.

[70] Brattig NW (2004). The Major Surface Protein of Wolbachia Endosymbionts in Filarial Nematodes Elicits Immune Responses through TLR2 and TLR4. The Journal of Immunology.

[71] Oliveira AC (2010). Impaired innate immunity in Tlr4(−/−) mice but preserved CD8+ T cell responses against Trypanosoma cruzi in Tlr4-, Tlr2-, Tlr9- or Myd88-deficient mice. PLoS Pathog.

[72] Arai R, Ueda H, Kitayama A, Kamiya N, Nagamune T (2001). Design of the linkers which effectively separate domains of a bifunctional fusion protein. Protein Eng.

[73] Soboslay PT (1994). Ivermectin-facilitated immunity in onchocerciasis; activation of parasite-specific Th1-type responses with subclinical Onchocerca volvulus infection. Clin Exp Immunol.

[74] Hess JA (2016). The Immunomodulatory Role of Adjuvants in Vaccines Formulated with the Recombinant Antigens Ov-103 and Ov-RAL-2 against Onchocerca volvulus in Mice. PLOS Neglected Tropical Diseases.

[75] Lustigman S, James ER, Tawe W, Abraham D (2002). Towards a recombinant antigen vaccine against Onchocerca volvulus. Trends in Parasitology.

[76] Corradin G, Villard V, Kajava AV (2007). Protein structure based strategies for antigen discovery and vaccine development against malaria and other pathogens. Endocr Metab Immune Disord Drug Targets.

[77] Pfarr KM, Fischer K, Hoerauf A (2003). Involvement of Toll-like receptor 4 in the embryogenesis of the rodent filaria Litomosoides sigmodontis. Med Microbiol Immunol.

[78] Soboslay PT (1997). The diverse expression of immunity in humans at distinct states of Onchocerca volvulus infection. Immunology.

[79] Ekkens MJ (2007). Th1 and Th2 Cells Help CD8 T-Cell Responses. Infection and Immunity.

[80] Carvalho LH (2002). IL-4-secreting CD4+ T cells are crucial to the development of CD8+ T-cell responses against malaria liver stages. Nat Med.

[81] Huang H (2007). CD4(+) Th1 cells promote CD8(+) Tc1 cell survival, memory response, tumor localization and therapy by targeted delivery of interleukin 2 via acquired pMHC I complexes. Immunology.

[82] Ward DJ (1988). Onchocerciasis and immunity in humans: enhanced T cell responsiveness to parasite antigen in putatively immune individuals. J Infect Dis.

[83] Elson LH (1995). Immunity to onchocerciasis: putative immune persons produce a Th1-like response to Onchocerca volvulus. J Infect Dis.

[84] Anthony RM, Rutitzky LI, Urban JF, Stadecker MJ, Gause WC (2007). Protective immune mechanisms in helminth infection. Nat Rev Immunol.

[85] Gori A, Longhi R, Peri C, Colombo G (2013). Peptides for immunological purposes: design, strategies and applications. Amino Acids.

[86] Chen R (2012). Bacterial expression systems for recombinant protein production: E. coli and beyond. Biotechnol Adv.

[87] Rosano GL, Ceccarelli EA (2014). Recombinant protein expression in Escherichia coli: advances and challenges. Frontiers in Microbiology.

[88] Nielsen H (2017). Predicting Secretory Proteins with SignalP. Methods Mol Biol.

[89] Petersen TN, Brunak S, von Heijne G, Nielsen H (2011). SignalP 4.0: discriminating signal peptides from transmembrane regions. Nature Methods.

[90] Almagro Armenteros JJ, Sonderby CK, Sonderby SK, Nielsen H, Winther O (2017). DeepLoc: prediction of protein subcellular localization using deep learning. Bioinformatics.

[91] Jespersen, M. C., Peters, B., Nielsen, M. & Marcatili, P. BepiPred-2.0: improving sequence-based B-cell epitope prediction using conformational epitopes. *Nucleic Acids Res* (2017). 10.1093/nar/gkx346 PMC5570230 28472356

[92] Yao B, Zhang L, Liang S, Zhang C (2012). SVMTriP: A Method to Predict Antigenic Epitopes Using Support Vector Machine to Integrate Tri-Peptide Similarity and Propensity. PLOS ONE.

[93] El-Manzalawy Y, Dobbs D, Honavar V (2008). Predicting linear B-cell epitopes using string kernels. J Mol Recognit.

[94] Faria AR (2011). High-Throughput Analysis of Synthetic Peptides for the Immunodiagnosis of Canine Visceral Leishmaniasis. PLOS Neglected Tropical Diseases.

[95] Peters B, Bulik S, Tampe R, Van Endert PM, Holzhutter HG (2003). Identifying MHC class I epitopes by predicting the TAP transport efficiency of epitope precursors. J Immunol.

[96] Nielsen M, Lund O (2009). NN-align. An artificial neural network-based alignment algorithm for MHC class II peptide binding prediction. BMC Bioinformatics.

[97] Dhanda SK, Vir P, Raghava GP (2013). Designing of interferon-gamma inducing MHC class-II binders. Biology Direct.

[98] Magnan CN (2010). High-throughput prediction of protein antigenicity using protein microarray data. Bioinformatics.

[99] Doytchinova IA, Flower DR (2007). VaxiJen: a server for prediction of protective antigens, tumour antigens and subunit vaccines. BMC Bioinformatics.

[100] Dimitrov I, Bangov I, Flower DR, Doytchinova I (2014). AllerTOP v.2–a server for in silico prediction of allergens. J Mol Model.

[101] Dimitrov I, Naneva L, Doytchinova I, Bangov I (2014). AllergenFP: allergenicity prediction by descriptor fingerprints. Bioinformatics.

[102] Gasteiger, E. *et al*. Protein Identification and Analysis Tools on the ExPASy Server. In *The Proteomics Protocols Handbook* (ed. Walker, J. M.) 571–607 (Humana Press, Totowa, NJ, 2005).

[103] Smialowski P, Doose G, Torkler P, Kaufmann S, Frishman D (2012). PROSO II–a new method for protein solubility prediction. Febs j.

[104] Wang S, Peng J, Ma J, Xu J (2016). Protein Secondary Structure Prediction Using Deep Convolutional Neural Fields. Scientific Reports.

[105] Yang, Y. *et al*. Sixty-five years of the long march in protein secondary structure prediction: the final stretch? *Brief Bioinform* (2016). 10.1093/bib/bbw129 PMC5952956 28040746

[106] Roy A, Kucukural A, Zhang Y (2010). I-TASSER: a unified platform for automated protein structure and function prediction. Nature Protocols.

[107] I-TASSER, I.T.A.R. I-TASSER, Iterative Threading ASSEmbly Refinement. (2018, January 12).

[108] Xu D, Zhang Y (2011). Improving the physical realism and structural accuracy of protein models by a two-step atomic-level energy minimization. Biophys J.

[109] Heo L, Park H, Seok C (2013). GalaxyRefine: protein structure refinement driven by side-chain repacking. Nucleic Acids Research.

[110] Wiederstein M, Sippl MJ (2007). ProSA-web: interactive web service for the recognition of errors in three-dimensional structures of proteins. Nucleic Acids Research.

[111] Lovell SC (2003). Structure validation by Calpha geometry: phi, psi and Cbeta deviation. Proteins.

[112] Barlow DJ, Edwards MS, Thornton JM (1986). Continuous and discontinuous protein antigenic determinants. Nature.

[113] Van Regenmortel MHV (1996). Mapping Epitope Structure and Activity: From One-Dimensional Prediction to Four-Dimensional Description of Antigenic Specificity. Methods.

[114] Ponomarenko J (2008). ElliPro: a new structure-based tool for the prediction of antibody epitopes. BMC Bioinformatics.

[115] Binkowski TA, Naghibzadeh S, Liang J (2003). CASTp: Computed Atlas of Surface Topography of proteins. Nucleic Acids Research.

[116] Hise AG, Gillette-Ferguson I, Pearlman E (2003). Immunopathogenesis of Onchocerca volvulus keratitis (river blindness): a novel role for TLR4 and endosymbiotic Wolbachia bacteria. J Endotoxin Res.

[117] de Vries SJ, Bonvin AM (2011). CPORT: a consensus interface predictor and its performance in prediction-driven docking with HADDOCK. PLoS One.

[118] van Zundert GCP (2016). The HADDOCK2.2 Web Server: User-Friendly Integrative Modeling of Biomolecular Complexes. J Mol Biol.

[119] Wassenaar TA (2012). WeNMR: Structural Biology on the Grid. Journal of Grid Computing.

[120] Dominguez C, Boelens R, Bonvin AM (2003). HADDOCK: a protein-protein docking approach based on biochemical or biophysical information. J Am Chem Soc.

[121] Xue LC, Rodrigues JP, Kastritis PL, Bonvin AM, Vangone A (2016). PRODIGY: a web server for predicting the binding affinity of protein-protein complexes. Bioinformatics.

[122] Grote A (2005). JCat: a novel tool to adapt codon usage of a target gene to its potential expression host. Nucleic Acids Research.

[123] Morla S, Makhija A, Kumar S (2016). Synonymous codon usage pattern in glycoprotein gene of rabies virus. Gene.

[124] Rapin N, Lund O, Bernaschi M, Castiglione F (2010). Computational Immunology Meets Bioinformatics: The Use of Prediction Tools for Molecular Binding in the Simulation of the Immune System. PLOS ONE.

[125] (TOVA), T.O.V.f.A. 2020 vision of a vaccine against river blindness (2012).

